# Homoarginine/ADMA ratio and homoarginine/SDMA ratio as independent predictors of cardiovascular mortality and cardiovascular events in lower extremity arterial disease

**DOI:** 10.1038/s41598-018-32607-8

**Published:** 2018-09-21

**Authors:** Philipp Jud, Franz Hafner, Nicolas Verheyen, Andreas Meinitzer, Thomas Gary, Marianne Brodmann, Gerald Seinost, Gerald Hackl

**Affiliations:** 1 Division of Angiology, Department of Internal Medicine, Medical University Graz, Graz, Austria; 2 Division of Cardiology, Department of Internal Medicine, Medical University Graz, Graz, Austria; 3 Institute of Medical and Chemical Laboratory Diagnostics, Medical University Graz, Graz, Austria

## Abstract

Endothelial dysfunction plays a key role in development of atherosclerosis and lower extremity arterial disease (LEAD). Homoarginine, asymmetric dimethylarginine (ADMA) and symmetric dimethylarginine (SDMA) are sensitive markers for endothelial dysfunction and independent risk factors for cardiovascular death. However, homoarginine may influence the proatherogenic effects of ADMA and SDMA suggesting homoarginine/ADMA ratio or homoarginine/SDMA ratio as further predictors for cardiovascular mortality. Therefore, we investigated the predictive value of homoarginine/ADMA ratio and homoarginine/SDMA ratio related to cardiovascular mortality and cardiovascular events in claudicant patients with LEAD. 151 patients with intermittent claudication were included in a prospective observational study (observation time 7.7 ± 2.5 years) with cardiovascular mortality as main outcome parameter and the occurrence of cardiovascular events as secondary outcome parameter. Homoarginine, ADMA and SDMA were measured by high-performance liquid chromatography at baseline. Low homoarginine/ADMA ratio and homoarginine/SDMA ratio were independently associated with higher cardiovascular mortality (HR 2.803 [95% CI 1.178–6.674], p = 0.020; HR 2.782 [95% CI 1.061–7.290], p = 0.037, respectively) and higher incidence of cardiovascular events (HR 1.938 [95% CI 1.015–3.700], p = 0.045; HR 2.397 [95% CI 1.243–4.623], p = 0.009, respectively). We observed that homoarginine/ADMA ratio and homoarginine/SDMA ratio are independent predictors for long-term cardiovascular mortality and events in claudicant patients with LEAD.

## Introduction

Lower extremity arterial disease (LEAD) refers to atherosclerotic stenosis or occlusions of the arteries of the lower limbs. Well known risk factors for LEAD are smoking, arterial hypertension, hypercholesterolemia, and diabetes mellitus. The presence of LEAD is an indicator of generalized atherosclerosis and patients with LEAD have a significantly increased risk for potentially fatal cardiovascular events^1^.

Endothelial dysfunction plays a key role in development and clinical manifestation of atherosclerosis and consecutive cardiovascular diseases like LEAD, and represents an independent risk factor of cardiovascular morbidity and mortality^2^. There are several biochemical parameters indicating endothelial dysfunction including homoarginine, asymmetric dimethylarginine (ADMA) and symmetric dimethylarginine (SDMA). Homoarginine, a homologue of l-arginine, is a cationic amino acid which may increase the production of nitric-oxide (NO) by inhibiting arginase and protein arginine methyltransferases as well as serving as a substrate for NO synthases improving endothelial function on that way^3,4^. ADMA and SDMA are endogenous products of proteolysis of several cells including endothelial cells which may promote endothelial dysfunction. ADMA impedes directly the synthesis of NO by acting as a potent inhibitor of endothelial NO synthase while SDMA inhibits the cellular uptake of the NO precursor arginine acting not as an inhibitor of NO synthases^4,5^.

Accumulating evidence indicates that higher levels of ADMA and SDMA as well as lower levels of homoarginine seem to be independent risk factors for cardiovascular death with a direct association of the occurrence of cardiovascular events^6–9^. However, the correlation of homoarginine, ADMA and SDMA among each other is still discrepant^10–13^.

These controversial findings suggest that other factors may influence the effect of homoarginine, ADMA and SDMA on endothelial dysfunction and atherosclerosis. Homoarginine inhibit protein arginine methyltransferases, an enzyme generating ADMA, and may reduce consequently the levels of ADMA^4^. Additionally, the bioavailability of NO is modulated by both homoarginine and ADMA due to an intracellular competition for NO-synthase^14^. Due to this modulating effect of homoarginine on ADMA, homoarginine/ADMA ratio may be an interesting indicator for endothelial dysfunction and atherosclerosis. In fact, there are studies indicating an association between l-arginine/ADMA ratio or homoarginine/ADMA ratio and atherosclerotic diseases or mortality^15–18^ while data for l-arginine/SDMA ratio or homoarginine/SDMA are however still missing, probably because no obvious influence between homoarginine or l-arginine and SDMA is yet known.

Therefore, the aim of the present study was to investigate if homoarginine/ADMA ratio or homoarginine/SDMA ratio are independent predictors of cardiovascular mortality and cardiovascular events in claudicant patients with LEAD. Additionally, we want to investigate if low homoarginine/ADMA ratio and homoarginine/SDMA ratio may be associated with the occurrence of cardiovascular events as secondary outcome parameters.

## Results

ADMA, SDMA and homoarginine showed a right-skewed distribution and were therefore 10-logarithmized before use in parametrical statistical procedures. A total of 151 patients were included in the analysis. Cardiovascular death and cardiovascular events were recorded during a mean follow-up of 7.7 (±2.5) years. 49 patients died (all of them due to cardiovascular death) and 61 patients suffered cardiovascular events (40 myocardial infarctions, 21 strokes) during this follow-up time. Patients’ baseline characteristics are shown in Table 1.

**Table 1:** Patients’ characteristics at baseline.

n	151
Men, n (%)	101 (66.9)
Age (y), median (25^th^–75^th^ percentile)	67 (60–76)
BMI in kg/m^2^, mean (+/− standard deviation)	26.7 (±3.1)
Observation time in years, mean (+/− standard deviation)	7.7 (±2.5)
Previous history, n (%)	
Myocardial infarction	24 (15.9)
Cerebrovascular disease (stroke, TIA)	21 (13.9)
Diabetes mellitus	50 (33.1)
Arterial hypertension	122 (80.8)
Smoking	109 (72.2)
Current	56 (37.1)
Ex	53 (35.1)
Hypercholesterolemia	78 (51.7)
Kidney function	
eGFR in ml/min^−1^ 1.73 m^−2^, median (25^th^–75^th^ percentile)	62 (48–83)
Creatinine in mg/dl, median (25^th^–75^th^ percentile)	1.1 (1.0–1.3)
ABI, median (25^th^–75^th^ percentile)	0.69 (0.54–0.89)
Drug therapies at discharge, n (%)	
Antiplatelet agents	142 (94.1)
Beta-blockers	46 (30.5)
ACE inhibitors or ARBs	95 (62.9)
Statins	73 (48.3)
Hemoglobin in g/dl, median (25^th^–75^th^ percentile)	13.9 (12.8–15.1)
Platelets in g/l, median (25^th^–75^th^ percentile)	233 (188–269)
CRP in mg/l, median (25^th^–75^th^ percentile)	4 (2.9–9.0)
Lipids in mg/dl, median (25^th^–75^th^ percentile)	
LDL	119 (94–146)
HDL	46 (40–60)
Triglycerides	141 (104–204)
Lipoprotein (a)	13 (10–48)
HbA1c in %, median (25^th^–75^th^ percentile)	5.8 (5.4–6.5)
Compounds and ratios median (25^th^–75^th^ percentile)	
Homoarginine in µmol/L	1.58 (1.13–1.98)
ADMA in µmol/L	0.72 (0.67–0.77)
SDMA in µmol/L	0.70 (0.62–0.89)
Homoarginine/ADMA ratio	2.05 (1.56–2.98)
Homoarginine/SDMA ratio	2.13 (1.42–2.92)

Abbreviations: BMI: body mass index, TIA: transient ischemic attack, eGFR: estimated glomerular filtration rate, ABI: ankle-brachial index, ACE: inhibitors angiotensin-converting enzyme inhibitors, ARB: angiotensin-receptor blockers, CRP: C-reactive protein, LDL: low-density lipoproteins, HDL: high-density lipoproteins, HbA1c: hemoglobin A1c, ADMA: asymmetric dimethylarginine, SDMA: symmetric dimethylarginine.

**Figure 1:**
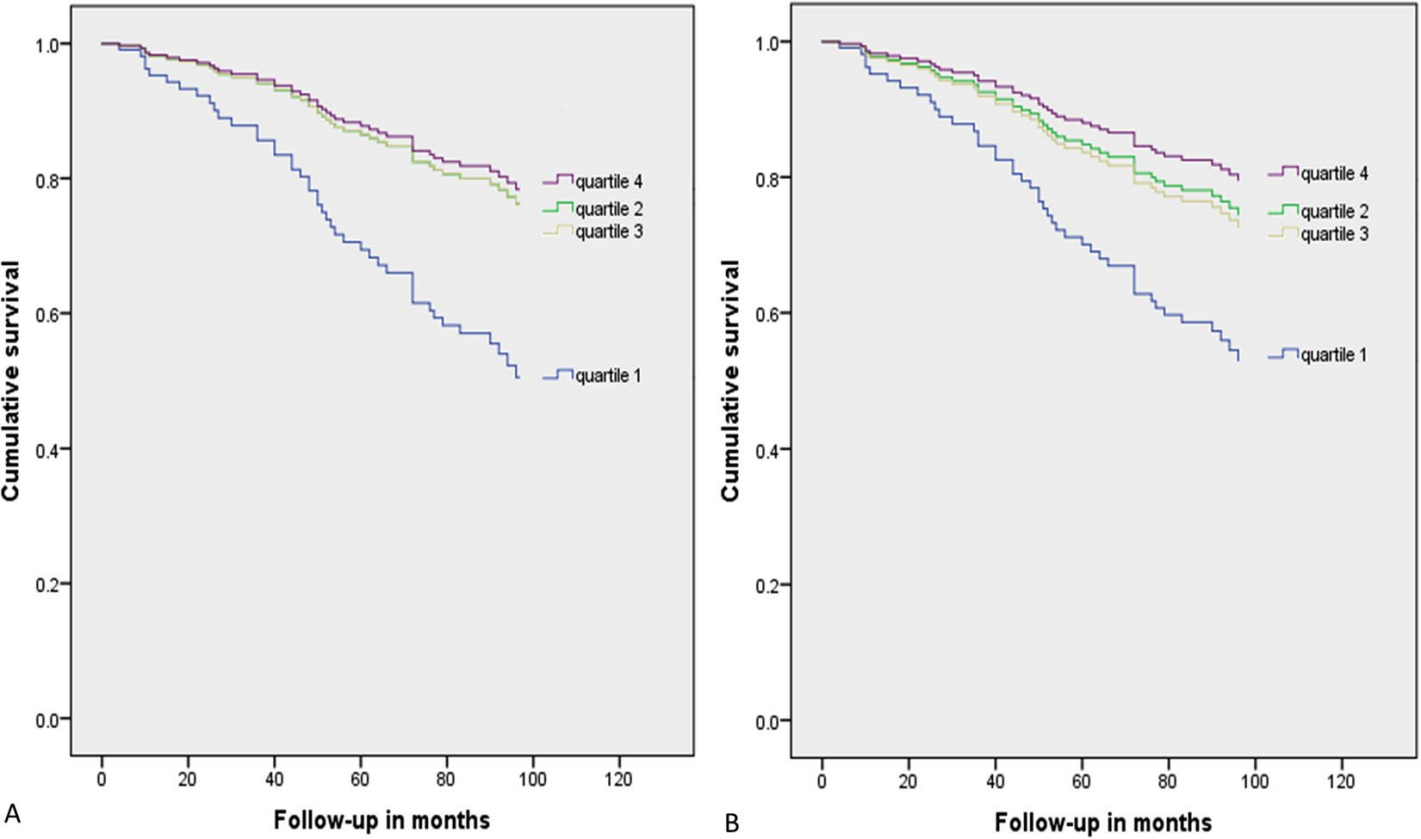
Kaplan-Meier curves for survival of cardiovascular death between quartiles of homoarginine/ADMA ratio (**A**) and homoarginine/SDMA ratio (**B**). In (**A**) the lines for quartile 2 and quartile 3 are almost super-imposed, due to almost identical hazard ratios and confidence intervals of both quartile 2 (HR [95% CI]: 1.115 [0.433–2.871]) and quartile 3 (HR [95% CI]: 1.113 [0.397–3.117]).

In univariate analyses, the lowest quartiles of both homoarginine/ADMA ratio and homoarginine/SDMA ratio were associated with a significantly increased risk of cardiovascular death when compared to the highest quartiles, respectively (HR 3.012 [95% CI 1.316–6.892], p = 0.009; HR 4.118 [95% CI 1.633–10.383], p = 0.003) (Fig. 1A,B). In multivariate analyses, the lowest quartile of homoarginine/ADMA ratio was also associated with a significantly increased risk of cardiovascular death when compared to the highest, respectively (HR 2.803 [95% CI 1.178–6.674], p = 0.020) (Table 2). Results were materially unchanged when adjusting for ADMA in quartiles or when adding diabetes, past myocardial infarction or smoking as covariates. Similarly, the lowest quartile of homoarginine/SDMA ratio was associated with a significantly increased risk of cardiovascular death when compared to the highest quartile in multivariate analyses (HR 2.782 [95% CI 1.061–7.290], p = 0.037) (Table 2). Results were also materially unchanged when adjusting for SDMA in quartiles or when adding diabetes, past myocardial infarction or smoking as covariates.

**Table 2:** Associations between homoarginine/ADMA ratio, homoarginine/SDMA ratio and cardiovascular death.

Dependent variable	Univariate analyses			Multivariate analyses		
	HR	95% CI	P-Value	HR	95% CI	P-Value
Homoarginine/ADMA	3.012	1.316–6.892	0.009	2.803	1.178–6.674	0.020
Homoarginine/SDMA	4.118	1.633–10.383	0.003	2.782	1.061–7.290	0.037

Associations were calculated using univariate and multivariate Cox regression analyses comparing the lowest with the highest quartile, respectively. Multivariate analyses were adjusted for age, sex, creatinine clearance and body mass index.

Abbreviations: HR: hazard ratio, CI: confidence interval.

Additionally, both homoarginine/ADMA ratio and homoarginine/SDMA ratio were associated with a significantly increased risk suffering cardiovascular events in univariate analyses when comparing the lowest to the highest quartile (HR 1.938 [95% CI 1.015–3.700], p = 0.045; HR 2.397 [95% CI 1.243–4.623], p = 0.009). However, there was no significant association in multivariate analyses.

**Figure 2:**
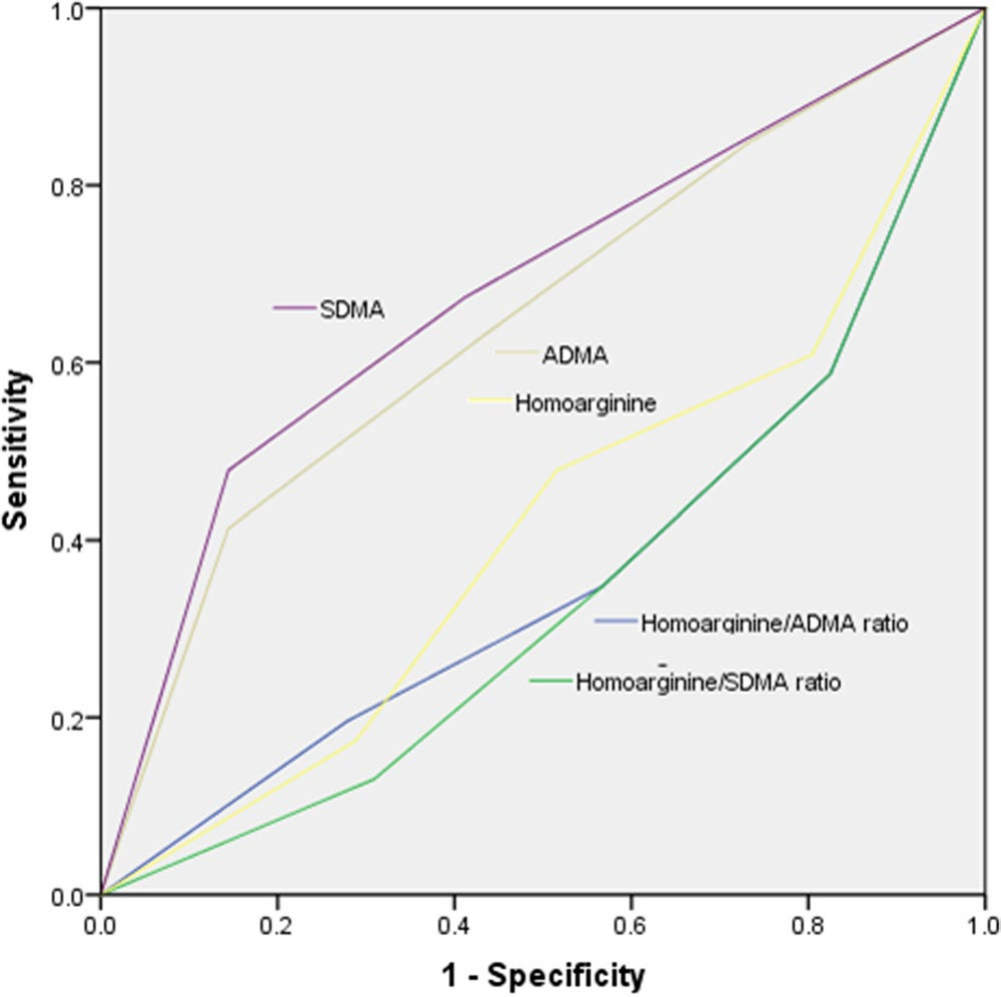
ROC curves for quartiles of homoarginine/ADMA ratio, homoarginine/SDMA ratio, homoarginine, ADMA and SDMA to predict cardiovascular death. AUC (95% confidence intervals) were 0.365 (0.264–0.466) for homoarginine/ADMA ratio, 0.341 (0.246–0.437) for homoarginine/SDMA ratio, 0.649 (0.549–0.749) for ADMA, 0.681 (0.583–0.780) for SDMA and 0.413 (0.312–0.515) for homoarginine.

ADMA was significantly associated with cardiovascular death in multivariate analyses (HR 2.753 [95% CI 1.158–6.541], p = 0.022), while SDMA was not (HR 1.729 [95% CI 0.693–4.311], p = 0.241). Table 3 summarizes the associations between ADMA, SDMA and homoarginine with cardiovascular death. Figure 2 depicts receiver operating curves (ROC) for quartiles of homoarginine/ADMA ratio, homoarginine/SDMA ratio, homoarginine, ADMA and SDMA as predictors of cardiovascular death.

**Table 3:** Associations between homoarginine, ADMA and SDMA and cardiovascular death, respectively.

Dependent variable	Univariate analyses			Multivariate analyses		
	HR	95% CI	P-Value	HR	95% CI	P-Value
Homoarginine	0.307	0.128–0.736	0.008	0.318	0.129–0.788	0.013
ADMA	3.206	1.411–7.284	0.005	2.753	1.158–6.541	0.022
SDMA	3.367	1.490–7.609	0.004	1.729	0.693–4.311	0.241

Associations were calculated using univariate and multivariate Cox regression analyses comparing the highest with the lowest quartile, respectively. Multivariate analyses were adjusted for age, sex, creatinine clearance and body mass index.

Abbreviations: HR: hazard ratio, CI: confidence interval.

## Discussion

In this prospective observational study, we demonstrated that homoarginine/ADMA ratio and homoarginine/SDMA ratio were inversely associated with cardiovascular death and independent predictors for cardiovascular mortality in claudicant patients with LEAD. In addition, homoarginine/ADMA ratio and homoarginine/SDMA ratio were independent predictors for the occurrence of cardiovascular events in univariate analyses, although both ratios did not achieve statistical significance in multivariate analyses which may be due to the rather small and selected study population. Interestingly, although the hazard ratios for cardiovascular death of homoarginine/ADMA ratio and homoarginine/SDMA ratio (2.803; 2.782, respectively) were similar or even higher compared to the hazard ratios of ADMA and SDMA (2.758; 1.729) in multivariate analyses, the area under the curve (AUC) in ROC analyses of ADMA and SDMA (0.649; 0.681), respectively) were better than the AUC of homoarginine/ADMA ratio and homoarginine/SDMA ratio (0.365; 0.341, respectively). It seems that homoarginine/ADMA ratio and homoarginine/SDMA ratio are novel biomarkers for cardiovascular death in claudicant patients with LEAD, but do not outperform ADMA and SDMA, at least in our analyses. Further evaluation in other studies are needed to clarify this finding.

Due to a modulating effect of homoarginine on ADMA given by the homoarginine/ADMA ratio^14^, it seems to make sense providing a homoarginine/ADMA ratio in patients with LEAD. Homoarginine and ADMA have been considered to be independent risk factors for cardiovascular mortality by influencing the NO signaling^6–9^. While there is a large number of data on the clinical roles of homoarginine and ADMA, it is less known about the biochemical roles of these two compounds, especially the interactions between each other and between other compounds of the homoarginine and arginine metabolism like SDMA, citrulline and ornithine. The homoarginine/arginine metabolism including its different compounds and enzymes can be viewed rather as a complicated biochemical system with many interacting pathways than just a single, simple pathway^4,14,15^. Therefore, different compounds including homoarginine, ADMA and SDMA may modulate their effects each other in this complicated metabolism and these regulative effects may be better represented by providing ratios of contrarily effecting compounds than levels of the single parameters. The fact, that also a homoarginine/SDMA ratio is an independent predictor, at least in our patient cohort, is an interesting novel finding. SDMA causing a decrease of NO by inhibition of the cellular uptake of arginine^5^ without any currently known molecular pathway or modulating effect how homoarginine may influence SDMA or vice versa. Additionally, homoarginine/SDMA ratio is not yet reported in literature, probably due to the assumption that homoarginine and SDMA have no modulating effect between each other. Further investigations are urgently needed to explore potentially unknown biochemical pathways of homoarginine, ADMA and SDMA and their potentially modulating effect as well as to investigate if the homoarginine/SDMA ratio is an appropriate predictor for cardiovascular death in patients with other cardiovascular diseases.

Bode-Böger *et al*.^15^. implicated with the ‘L-arginine paradox’ the importance of the l-arginine/ADMA ratio and several studies demonstrated that lower l-arginine/ADMA ratio was a risk factor for all-cause mortality and atherosclerosis^16,17^. Additionally, also low homoarginine/ADMA ratio correlated significantly with aortic intima-media thickness in stroke patients^18^. Nevertheless, it must be noted that most studies investigating an ‘arginine/ADMA ratio’ measured l-arginine and not homoarginine. While l-arginine and homoarginine are biochemically familiar, their clinical data are discrepant or even contrary. Clinical data about homoarginine seem to be more conclusively than l-arginine so that we decided to measure homoarginine instead of l-arginine in this study. Low plasma levels of homoarginine are independently associated with high cardiovascular and all-cause mortality and high levels of homoarginine have a protective effect on the cardiovascular system^4,9,19,20^. In contrast, high levels of l-arginine may be associated with progression of atherosclerosis as well as cardiovascular diseases, and additionally supplementation of l-arginine had no beneficial effect on cardiovascular diseases or may be even harmful^4,10,21–23^. However, it must be noted that data about supplementation of homoarginine in a secondary or tertiary prevention setting, especially for patients with LEAD, are not yet available. Therefore, it is currently not known if homoarginine supplementation would have beneficial effects on cardiovascular diseases. On the other hand, the biochemical metabolism of l-arginine is more extensively investigated than of homoarginine. While l-arginine acts as a potent substrate for NOS improving on that way endothelial function, the underlying biochemical pathways of the homoarginine metabolism are less investigated and cannot fully elucidate the clinical results^4,24^. Because homoarginine is a rather poor substrate for NOS compared to l-arginine, a direct effect of homoarginine on NOS catalysis may be unlikely, and additionally it is still unclear if higher levels of homoarginine would increase directly NO synthesis^4,24^. Another potential mechanism whereby homoarginine may exert its beneficial cardiovascular effects may be due to inhibition of the enzyme arginase^4^. However, Tommasi *et al*.^25^. recently found that the inhibitory effect of homoarginine at physiological concentrations on arginase 1 and 2 seems to be too low to achieve the beneficial cardiovascular effects suggesting that a homoarginine-mediated arginase inhibition is unlikely to be a key mechanism for the beneficial cardiovascular effects of homoarginine. As mentioned above, further studies investigating homoarginine metabolism and especially clinical trials using homoarginine supplementation need to be performed.

There are limitations of this study. The number of participants is rather small so that particularly our negative findings may be the result of a type 2 error and should be regarded with caution. Moreover, we did not include patients with other stages of LEAD, but on the other hand, this study was conducted with a very selective patient cohort (claudicant patients with LEAD Rutherford classification 2–3 prior to the first endovascular intervention). Nevertheless, both, homoarginine/ADMA ratio and homoarginine/SDMA ratio, emphasize their important predictive value in claudicant patients with LEAD, while consequently a larger population of claudicant patients with LEAD and also a population of patients with other stages of LEAD are necessary.

In conclusion, the present study suggests that low homoarginine/ADMA ratio as well as homoarginine/SDMA ratio were independent predictors for cardiovascular mortality and cardiovascular events in claudicant patients with LEAD.

## Methods

### Study design and subjects

Patients with intermittent claudication (Rutherford classification stage 2–3), who underwent their first endovascular procedure of the pelvic and/or femoropopliteal arteries, were screened for study inclusion in this prospective observational study. Finally, a total of 151 consecutive patients (50 female, 101 male) were included in this study with cardiovascular related death as the primary outcome parameter and occurrence of cardiovascular events as secondary outcome parameter. The occurrence of fatal stroke or fatal myocardial infarction was defined as cardiovascular related death. Cardiovascular events were defined as the occurrence of fatal and non-fatal stroke as well as fatal or non-fatal myocardial infarction. Patients suffering from unstable angina pectoris, acute myocardial infarction or stroke at the time of recruitment were excluded. Additionally, patients suffering from LEAD below the knee objectified by arterial Doppler ultrasonography or magnetic resonance angiography were excluded as intermittent claudication (Rutherford classification stage 2–3) is not an indication for endovascular recanalization in such patients^26^. Other exclusion criteria were uncontrolled hypertension, decompensated heart failure, life expectancy of less than one year due to a not curable disease, wound infections, and contraindications against anticoagulants and/or antiplatelet agents. Patients were also excluded who died due to a non-cardiovascular cause.

### Data collection

Patients’ baseline characteristics were determined on the day of the endovascular intervention. Subsequently, a total of four follow-up visits after 1 month, 3 months, 6 months, and 12 months were scheduled. At each study visit, the occurrence of cardiovascular death, cardiovascular events, and patients’ concomitant medication were recorded. Between October 2010 and May 2011, all patients were contacted by the investigators to document the occurrence of cardiovascular events. For this purpose, patients were invited to an outpatient examination/survey, in which they answered questions about their LEAD symptoms, medical history, and current medication. Using the same survey, patients who could not participate in the examination were interviewed via telephone as an alternative means of data collection. If patients were deceased or not reachable by telephone, the primary care physician of the respective patients was contacted to provide the information, which included cardiovascular endpoints such as death, cause of death, stroke, and myocardial infarction. That enabled the collection of the necessary data regarding mortality, cardiovascular events, as well as current medication. Finally, all medical files in all public Styrian hospitals including their emergency departments and divisions of pathology were reviewed in order to complete data collection.

### Biochemical analyses

At the baseline visit on the day of the endovascular intervention, fasting blood samples were obtained. The serum was centrifuged and stored at −70 °C until further analysis of homoarginine, ADMA and SDMA were performed in March 2011 by means of high-performance liquid chromatography with a solid phase extraction and precolumn derivatization technique which was first described by Teerlink with only minor modifications^27,28^. According to previous reports, the investigated biomarkers can be assumed as stable^29^. Within-day coefficients of variation for homoarginine were 4.7% (1.21 µmol/L) and 2.2% (3.53 µmol/L), and between-day coefficients of variation were 7.9% (1.25 µmol/L) and 6.8% (3.66 µmol/L). Within-day coefficients of variation for ADMA were 3.1% (0.62 µmol/L) and 1.0% (2.0 µmol/L), and between-day coefficients of variation were 9.0% (0.62 µmol/L) and 2.2% (2.0 µmol/L). Within-day coefficients of variation for SDMA were 4.6% (0.60 µmol/L) and 1.9% (1.0 µmol/L), and between-day coefficients of variation were 9.8% (0.60 µmol/L) and 6.1% (1.0 µmol/L).

### Statistics

In case of continuous variables, patient characteristics were given as means (±standard deviation). Median and interquartile range were used to express skewed data. Categorical variables were represented by frequency and percentages. The normal distribution was examined via Kolmogorov-Smirnov test. Non-normally distributed variables were 10-logarithmically transformed before use in parametrical statistical procedures. The two-sided t test was used for the comparison of groups in case of parametrical distribution. For non-parametrical data, a Mann-Whitney-U test was utilized. Univariate and multivariate cox regression hazard models were performed to assess the homoarginine/ADMA ratio, the homoarginine/SDMA ratio, as predictors of both cardiovascular death and cardiovascular events and comparing the highest with the lowest quartile, respectively. We also assessed ADMA, SDMA and homoarginine as predictors of cardiovascular death comparing the lowest with the highest quartile, respectively. Analyses were adjusted for important confounding variables, including age, sex, creatinine clearance and body mass index aiming to include one covariate per 10 events. In further analyses, we included also diabetes, smoking (yes/no) and past myocardial infarction as additional covariates.

The accuracy of quartiles of homoarginine/ADMA ratio, homoarginine/SDMA ratio, homoarginine, ADMA and SDMA to predict cardiovascular death were calculated using ROC area under the curve (AUC) analyses, with cardiovascular death as the state variable.

Assuming a Cox proportional HR of 3.0 comparing quartile 1 with quartile 4, an event rate of 50 was considered sufficient to achieve a power of 80% at a significance level of 5%.

We assumed statistical significance when P-value was <0.05. Statistical analyses were executed via SPSS version 23.0.

### Ethical approval and informed consent

The study was approved by the Institutional Review Board of the Medical University Graz, Austria (EK 23-038 ex 10/11). We confirm that all research was carried out accordingly with the relevant guidelines and regulations and all patients gave their written informed consent after being accurately informed about the clinical trial.

## Data Availability

The datasets generated and analyzed during the current study are available from the corresponding author on reasonable request.
